# Visualizing the Residue Interaction Landscape of Proteins by Temporal Network Embedding

**DOI:** 10.1021/acs.jctc.2c01228

**Published:** 2023-04-26

**Authors:** Leon Franke, Christine Peter

**Affiliations:** † Department of Chemistry, University of Konstanz, Universitätsstraße 10, Konstanz 78457, Germany; ‡ Konstanz Research School Chemical Biology, University of Konstanz, Universitätsstraße 10, Konstanz 78457, Germany

## Abstract

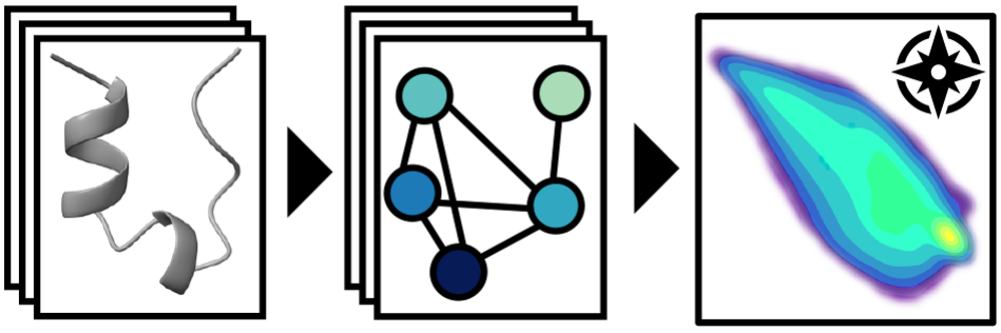

Characterizing the structural dynamics of proteins with heterogeneous conformational landscapes is crucial to understanding complex biomolecular processes. To this end, dimensionality reduction algorithms are used to produce low-dimensional embeddings of the high-dimensional conformational phase space. However, identifying a compact and informative set of input features for the embedding remains an ongoing challenge. Here, we propose to harness the power of Residue Interaction Networks (RINs) and their centrality measures, established tools to provide a graph theoretical view on molecular structure. Specifically, we combine the closeness centrality, which captures global features of the protein conformation at residue-wise resolution, with EncoderMap, a hybrid neural-network autoencoder/multidimensional-scaling like dimensionality reduction algorithm. We find that the resulting low-dimensional embedding is a meaningful visualization of the residue interaction landscape that resolves structural details of the protein behavior while retaining global interpretability. This feature-based graph embedding of temporal protein graphs makes it possible to apply the general descriptive power of RIN formalisms to the analysis of protein simulations of complex processes such as protein folding and multidomain interactions requiring no protein-specific input. We demonstrate this on simulations of the fast folding protein Trp-Cage and the multidomain signaling protein FAT10. Due to its generality and modularity, the presented approach can easily be transferred to other protein systems.

## 1 Introduction

Molecular dynamics (MD) simulations offer a powerful tool for studying increasingly complex biomolecular systems at a high temporal and spatial resolution as they evolve under the forces from classical mechanics. Advances in computational power and methods make it possible to produce ever-longer simulations of ever-larger systems.^1,2^ Extracting meaning from the resulting large and high-dimensional trajectories—time-ordered sequences of atom positions—is as challenging as producing the data in the first place. This is confounded by the fact that the conformational ensemble of a protein often comprises highly nonlinear, concerted dynamics on multiple scales. Large, global conformational motions of entire domains can be intertwined with subtle, local fluctuations in flexible regions of the protein. At both ends of that range, and in-between, these dynamics can critically determine the function of the protein.^3^ Finding a meaningful and interpretable representation of heterogeneous conformational ensembles at different scales is a crucial, yet challenging step in extracting insights from protein MD data sets.

For the visualization and downstream analysis of complex protein ensembles, low-dimensional maps of the protein’s conformational landscape are often indispensable. One can obtain such maps by hand-selecting specific scalar descriptors derived from the protein structure, for example, the root-mean-square deviation (RMSD) from a given reference conformation, the radius of gyration (*R*_*g*_) or the fraction of native contacts.^3,4^ Used as coordinates of a map, such collective variables (CVs) can provide a global view of protein dynamics, omitting details on a local scale, or, if specific atom distances or dihedral angles are selected as CVs, a bespoke, narrow view of local behavior can be obtained.^4^ If, on the other hand, multiple conformational processes contribute to the conformational ensemble, then it is often useful to take some high-dimensional numerical description of the protein structure and obtain a low-dimensional map or embedding of that description with the help of dimensionality reduction algorithms.^5,6^ One typically does not directly use the full set of Cartesian coordinates of the trajectory as the high-dimensional input descriptors. Instead, each protein conformation is translated into a feature vector in a process called featurization. The featurization step crucially determines which aspects of the underlying protein dynamics are reflected in the embedding and which are discarded.^6^ For example, one can focus on the local dynamics of the protein backbone by using its backbone dihedral angles as a feature set, discarding information on the side chain dynamics and global behaviors. Using pairwise distances metrics between amino acid side chains, such as contact matrices, one can retain more comprehensive details, yet the resulting data sets can become prohibitively large and often contain redundant information, which can necessitate the selection of a subset of pairwise distances one considers representative of relevant dynamics.^7^ The intermediate step of featurization thus offers the opportunity to adjust the scope of the analysis and put a focus on specific protein behaviors.^8^ However, this often means that it needs to be tailored to the protein system with system-specific additional input. This could be a native structure, for methods based on native contacts,^9^ or prior knowledge on the system dynamics for hand-selecting specific features of interest. For an embedding algorithm for complex protein dynamics that does not require protein-specific input or tailoring, we need a general protein description that captures the relevant conformational behavior of different protein systems in a compact and meaningful way.^6^

We can describe the structure of proteins in an expressive and general way by constructing a network from the interactions between their amino acid residues. These so-called residue interaction networks (RINs) contain nodes and edges, where the nodes represent the amino acid residues of the protein, and the connecting edges represent interactions or contacts between the residues. This focus on residue contacts provides a mesoscopic perspective on protein dynamics, connecting behaviors on a local and global scale.^10,11^ Leveraging the network theoretic methods that become accessible when describing proteins as networks, RINs have been employed in numerous studies to understand the structure and function of proteins.^12−16^ Often these studies focused on analyzing one or a few individual networks. These were either derived from static protein structures^17,18^ or generated by aggregating the frames from a full MD trajectory into a summary network description, e.g., by averaging the contacts^19^ or calculating correlations between residue motions.^20,21^ However, for protein systems with complex, multistate conformational ensembles, such a summary system representation, with one or a few networks may average over and thus fail to capture relevant aspects of the structural dynamics,^22^ resulting in a loss of temporal resolution. This is particularly true if the protein conformations cannot be easily grouped into one or a few states, for example, because the conformational ensemble of the protein in solution is highly heterogeneous, as is the case for protein folding simulations, or because there is no known native reference structure, like for intrinsically disordered proteins. Of course, merely replacing a single-graph description with a sequence or time series of graphs from a full MD trajectory will not yield a more interpretable picture of the system’s conformational dynamics.

In the present work, we want to combine the expressiveness and generality of the RIN formalism with the interpretability and dynamic resolution of a landscape embedding. The goal is to obtain an interpretable, temporal graph embedding for RINs from full-length MD trajectories of complex protein systems. By arranging the conformations of the protein in a two-dimensional map based on their residue interactions, we obtain a visualization of the protein’s “residue interaction landscape”. To extract a feature set from the RINs that can be used for the embedding, network theory opens up a straightforward avenue: Node centralities provide a mathematically rigorous formalism to capture the role of amino acid residues in protein structure and function.^12,18^ The maps resulting from a low-dimensional embedding of the nodes’ closeness centralities resolve meaningful details of the protein behaviors while retaining global structure and interpretability. The method can be applied to different protein systems without protein-specific input. We demonstrate this on the small and fast folding protein Trp-Cage^23^ and on the multidomain signaling protein FAT10.^24^

## 2 Results

### 2.1 Workflow for Visualizing Residue Interaction Landscapes

**Figure 1:**
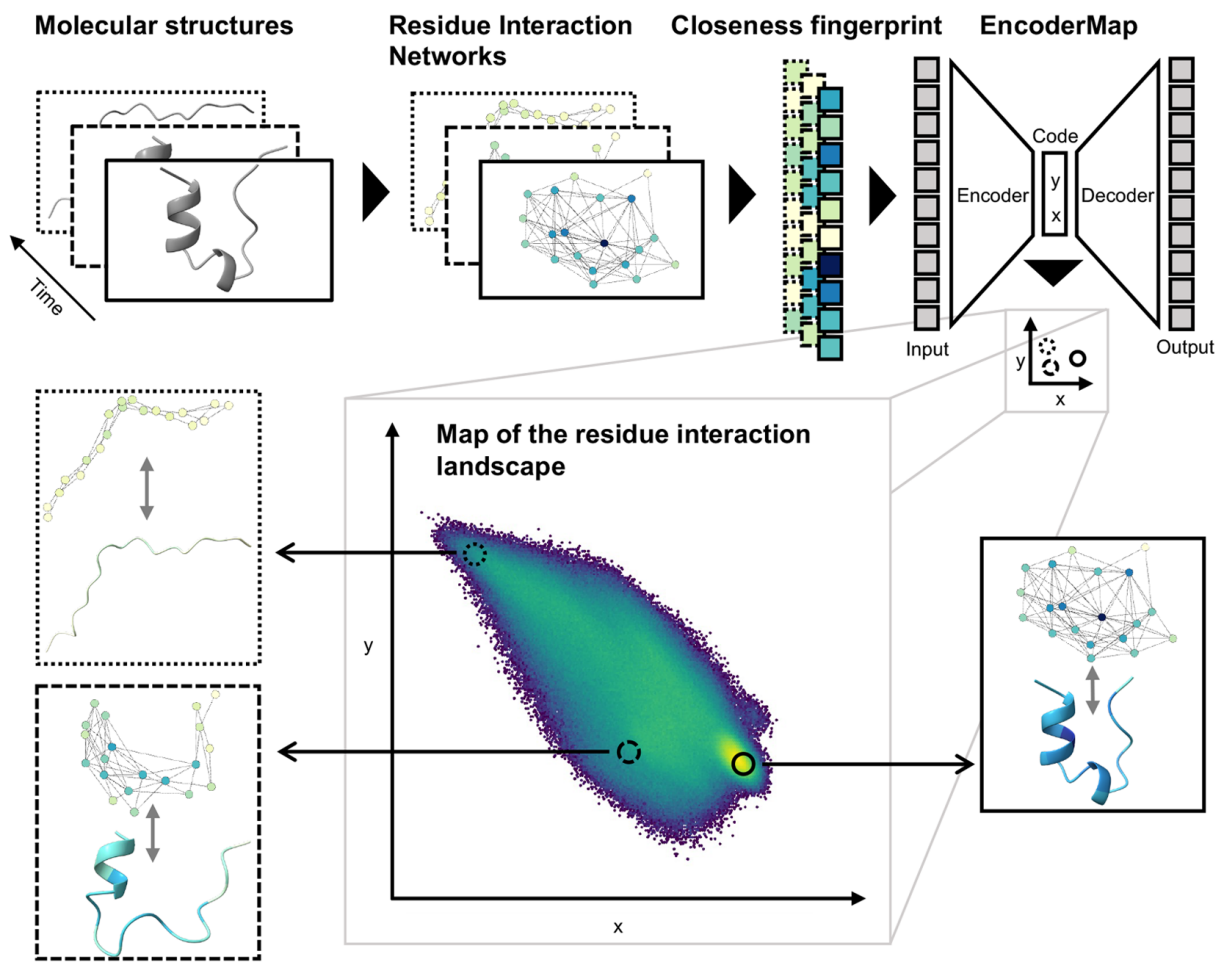
Visualizing protein residue interaction landscapes: Protein structures from molecular dynamics simulations were translated into residue interaction networks. From these, the closeness centrality of each residue is extracted as an *N*-dimensional feature set. An autoencoder-based dimensionality reduction (EncoderMap) is used to project this high-dimensional feature set into a low-dimensional embedding of the residue interaction networks, which resolves relevant protein behaviors.

The workflow for visualizing residue interaction landscapes is shown in Figure 1. In the first step, each frame from the simulation is translated into a residue interaction network, i.e., a network representing the protein structure. The nodes of the network represent the amino acid residues of the protein, and the edges represent interactions between them. There are many different formalisms to translate protein structures into RINs.^12,14−16^ Here, we are using a simple geometric criterion based on the spatial distance between residues. Two residues are connected by an edge if the minimum distance between any two atoms in the residues is below a cutoff of 6 Å, with the exception of interactions between directly neighboring residues in the protein sequence. This produces a discretized protein contact map, which is then translated into an undirected and unweighted RIN graph describing the residue interactions of an individual, static protein structure. From the RINs, we can calculate the node centrality of each residue based on various centrality metrics from network theory.^19,25−30^ Here, we propose to use the closeness centrality as a “natural” centrality metric,^31^ which describes how close or distant, in the network, each residue is to all other residues. In physical terms, this means that a residue’s closeness centrality is connected with that residue’s “aptitude to participate in signal transmission” throughout the RIN.^15^ It is calculated as the reciprocal mean length of the shortest paths from that node to all other nodes in the RIN according to , where *v*_*i*_ is the node of interest and  represents the length of the shortest geodesic path, an integer count of the number of edges, from *v*_*i*_ to any other node *v*_*j*_.^31^*N* is the total number of nodes/residues in the network, used here for normalization. It has been shown that the closeness centrality is an important network topological metric for predicting “functionally important residues”,^29^ such as protein–protein interaction sites,^27^ catalytic sites,^28^ and binding sites.^29,30^ Here, rather than focusing on the role of individual residues, the *N* closeness centralities, in the order of the protein’s amino acid sequence, are used as an *N*-dimensional fingerprint describing the connectivity of the graph representing the RIN. Because the closeness centrality for each residue takes into account network paths across the entire RIN, it integrates global information on the conformation of the protein and local information on the residue’s neighborhood.^15,29,30,32^ With *N* dimensions, it is still too high-dimensional to visualize. To project the high-dimensional feature vector into a low-dimensional map, we employ the dimensionality reduction algorithm EncoderMap, which couples a neural network autoencoder with a multidimensional-scaling-like embedding based on pairwise distances between data points. This feature-based graph embedding uses the Euclidean distances in the high-dimensional metric space of the closeness fingerprints to place protein structures that are close in this feature space close to each other in the low-dimensional map. In addition, the distances are transformed using a sigmoid function in order to suppress the impact of small distances between very similar points and of very large distances that would be hard to reproduce accurately, a transformation first applied for multidimensional scaling such as the Sketch-map algorithm^33^ and adapted for EncoderMap. The overview of the workflow (Figure 1) shows one of the resulting maps, visualizing the residue interaction landscape of a protein. It separates unfolded (i.e., low-contact) states of the protein from folded (i.e., high-contact) states. High-contact states have a greater number of contacts distinguishing the structures, which means that the map resolves different high-contact states and the transitions toward them with an increasing resolution. The overview also shows the modularity of the workflow, which in principle allows different elements of the workflow to be exchanged.

### 2.2 Closeness Centralities and Residue Interaction Landscape of Trp-Cage

**Figure 2:**
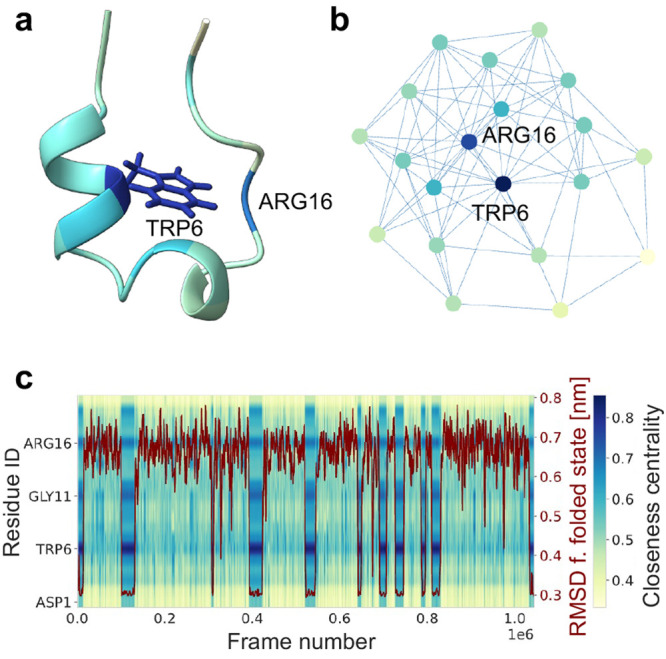
Fully folded, native structure of Trp-Cage (a) and corresponding RIN (b) drawn with a force-directed layout. Color corresponds to residue-wise closeness centrality (darker means higher, colorbar in c). The highlighted residues TRP6 and ARG16 are marked by a high closeness centrality. Both play an important role in Trp-Cage folding. (c) Closeness fingerprint over the time of the full simulation trajectory, overlaid with the RMSD from the folded state (red).

First, we demonstrate the approach on the well-characterized fast-folding model protein Trp-Cage. It has 20 residues and its native folded state consists of an N-terminal α helix (residues 2–8), a 3–10 helix, and an C-terminal proline helix, which cage a hydrophobic tryptophan residue (TRP6) in the center.^34^ The structure of Trp-Cage is shown in Figure 2a. The RIN corresponding to this structure is shown in Figure 2b, drawn with a force-directed graph layouting algorithm.^35^ Both are colored by the closeness fingerprint. The topology of the RIN and the closeness fingerprint reflect the structural features of Trp-Cage: The central TRP6 is in the center of the RIN and has the highest closeness centrality. Other residues are marked by a high closeness centrality as well, such as the ARG16, which participates in a salt bridge in the native folded state and in folding intermediates.^36,37^ Here, we analyze the ultralong simulation of Trp-Cage performed by D. E. Shaw research,^23^ a 208 μs long simulation trajectory with over 1,000,000 simulation frames. In Figure 2c, the temporal evolution of the closeness centrality of all residues is shown over the whole simulation trajectory. It is overlaid with the RMSD from the native, fully folded state (PDB 2JOF,^38^Figure 2c, red). We can follow the overall closeness centrality of all residues and distinguish nonfolded states from the folded state with a characteristic closeness centrality fingerprint. We can also follow the closeness centralities of individual residues, such as TRP6 and ARG16, and recognize their central role for the folded state and nonfolded intermediates. This shows that the closeness centrality is a feature set that has residue level resolution but also captures global protein behavior based on the residue contacts.

**Figure 3:**
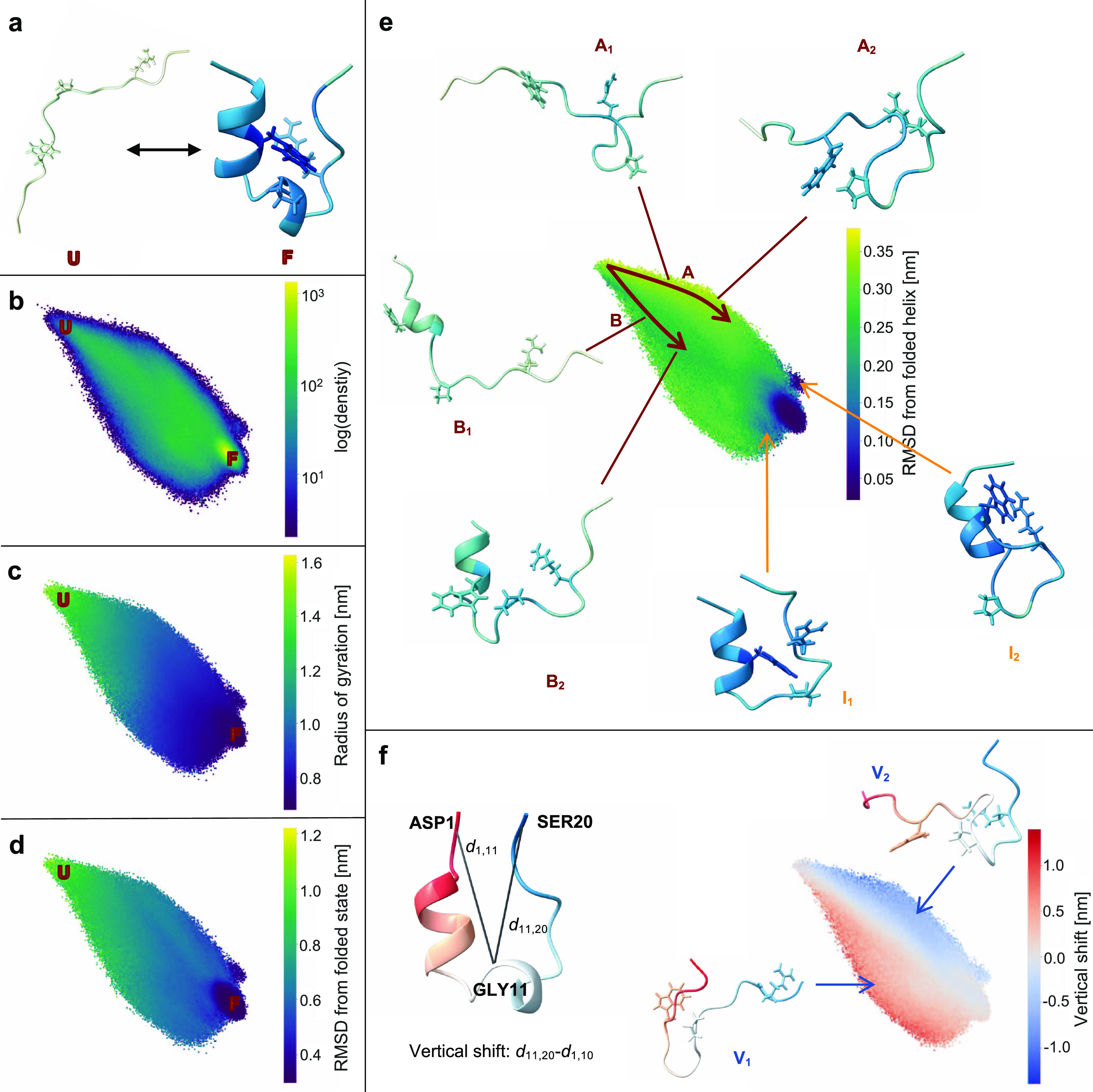
(a) Trp-Cage: fully unfolded (U) vs fully folded (F), some residues are drawn as sticks for orientation. Map of residue interaction landscape of Trp-Cage colored by density (b), radius of gyration (c), and RMSD from folded state (d) with U and F marked in the map. (e) Map of residue interaction landscape colored by helix-RMSD with representative conformations colored by closeness centrality: two separate folding transitions are highlighted in red: pathway A, nucleation-condensation; pathway B, diffusion-collision, two near-native folding intermediates are shown in orange (I_1_, I_2_). (f) Left: Illustration of vertical shift, an important CV to characterize Trp-Cage, difference between the distance from ASP1 to GLY11 and distance from GLY11 to SER20. Right: Map colored by vertical shift with representative conformations with high (V_1_) and low (V_2_) vertical shift, colored by residue ID.

To obtain a low-dimensional visualization of the conformational behavior of Trp-Cage, we projected each frame from the simulation trajectory into a low-dimensional embedding of its residue interaction landscape. The resulting map is shown in Figure 3. It separates fully unfolded, low-contact states (Figure 3aU) from the more compact, high-contact states along its diagonal axis from the top left to the bottom right. The maps are colored by the density and by several known collective variables commonly applied to describe and analyze the folding of Trp-Cage.^36,39−42^ Coloring the maps using these observables shows that they capture important, known features of this process Figure 3(b–d). The map colored by density (Figure 3b) shows that the folded state Figure 3aF forms a deep well in the residue interaction landscape, while the region with nonfolded conformations remains relatively featureless, an observation that has been made in other descriptions of Trp-Cage folding.^43^ The transition region between the far ends of the map resolves the folding transitions from fully unfolded (low-contact) conformations to higher-contact conformations with an increasing similarity to the folded state. This is reflected in the smooth gradient of the radius of gyration (*R*_*g*_) (Figure 3c) and the RMSD from the folded state (Figure 3d) along the first diagonal axis of the map. Along its second diagonal axis, more subtle details of the folding process are resolved. The high-contact region around the native state distinguishes near-native folding intermediates (Figure 3e I_1_, I_2_), which bear similarity to previously described folding intermediates.^36,39,41^ Coloration according to the helix-RMSD from the N-terminal α helix (residues 2–8) in Figure 3e shows that the map can distinguish two folding transitions in accordance with previous analyses of Trp-Cage folding:^36,39,44^ The nucleation-condensation pathway (Figure 3eA), in which a hydrophobic collapse forms the first step in folding, followed by the formation of the native fold. In the diffusion collision pathway (Figure 3eB), the N-terminal α-helix forms first, followed by the formation of the remaining secondary structure elements. In Figure 3f, the map is colored by the vertical shift of Trp-Cage, which is a measure for the relative position of the termini with respect to the center of the molecule. It was defined by Chen et al.^45^ as the difference between the distance *d*_1–11_ and the distance *d*_11–20_. It has been shown to be an important CV for Trp-Cage and is closely connected to the helix RMSD. The second diagonal of the residue interaction landscape is closely associated with this important descriptor of Trp-Cage.

We have shown that the map of the residue interaction landscape of Trp-Cage recovers meaningful descriptors of Trp-Cage’s conformational behavior, resolving relevant details of its folding. Together with its continuous global structure, this makes the map a meaningful and interpretable visualization of the conformational behavior of this small, fast-folding protein. Since the approach was applied without specific prior information about the protein or its folding characteristics, it should in principle be generalizable. This makes it particularly interesting for complex protein systems that do not have a known native reference conformation or little available structural information. An example of such a case are proteins that adopt a dynamic conformational ensemble with multiple metastable substates and changing intramolecular interactions. This could be because their function depends on flexible, disordered regions in their structure or because they have multiple domains whose interactions change dynamically.

### 2.3 Closeness Centralities and Residue Interaction Landscape of FAT10

**Figure 4:**
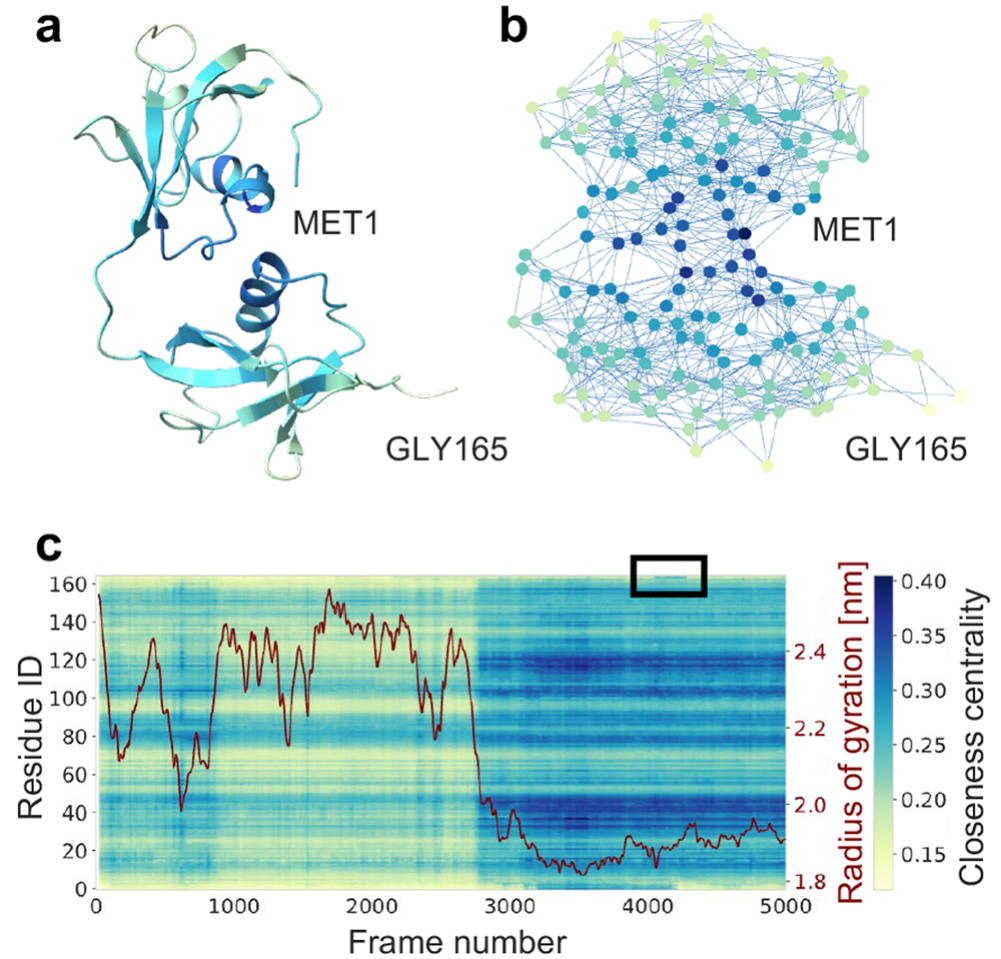
Closed conformation of FAT10 (a) and corresponding RIN (b), drawn with a force-directed layout. Color corresponds to the closeness centrality of each residue (darker means higher, colorbar in c), N- and C-terminus are highlighted for orientation. (c) Closeness fingerprint over one simulation trajectory, the radius of gyration is overlaid in red, and the black box highlights a region in which the C-terminal tail temproarily interacts with the rest of FAT10.

A protein that exhibits both functionally relevant flexible regions and changing domain–domain interactions is the ubiquitin-like modifier FAT10 (human leukocyte antigen (HLA)-F adjacent transcript 10). FAT10 is a 165-residue signaling protein that consists of two ubiquitin-like domains joined by a flexible linker.^46^ Both the N-terminus and the C-terminus have flexible tails. FAT10 gets isopeptide-linked via its C-terminal diglycine motif to its substrate proteins and labels them for degradation by the 26S proteasome.^24^ Therefore, it plays a role in cellular signaling and proteostasis and is associated with numerous types of cancer^47^ and Parkinson’s disease.^48^ The conformational ensemble of FAT10 crucially determines its function, i.e., interactions with substrates and the proteasome, and has thus far not been characterized in detail. This includes the interactions between the two domains and the dynamics of the flexible regions of FAT10: the N-terminal tail, the C-terminal tail and the linker.^24^ Here, we analyzed 50 × 50 ns of atomistic simulations of FAT10, corresponding to 250, 050 simulation frames. Each of the 50 simulation trajectories was started in an “open” conformation, in which the two domains were separated and had no nonconvalent contacts with each other. In the course of the simulations, the two domains would commonly collapse onto each other, forming “closed” conformations with different noncovalent contacts at the interface between them. A closed structure of FAT10 is shown in Figure 4a, along with its RIN description (Figure 4b), the residues are colored by their closeness centralities. Here, the RIN description also reflects the conformational characteristics, with the two separate domains, connected by the linker as well as noncovalent contacts. The closeness centralities of the residues are high at the domain interface. The time series of the closeness fingerprint of the residues from one 50 ns simulation (Figure 4c) allows for a detailed tracking of each of the residues behaviors. This includes local details, such as the behavior of the terminal tails. For example, the C-terminal tail, which is involved in substrate binding, is attached in the region marked by the black box and free otherwise. Viewed in total, the closeness fingerprint also shows whether FAT10 is in a low-contact (open) or in a high-contact (closed) state, as illustrated by the overlay of the *R*_*g*_ over the time series of the closeness fingerprint. In the Supporting Information (Figure S1), we further illustrate how the closeness fingerprint integrates information on the global protein conformation and the local residue environment.

**Figure 5:**
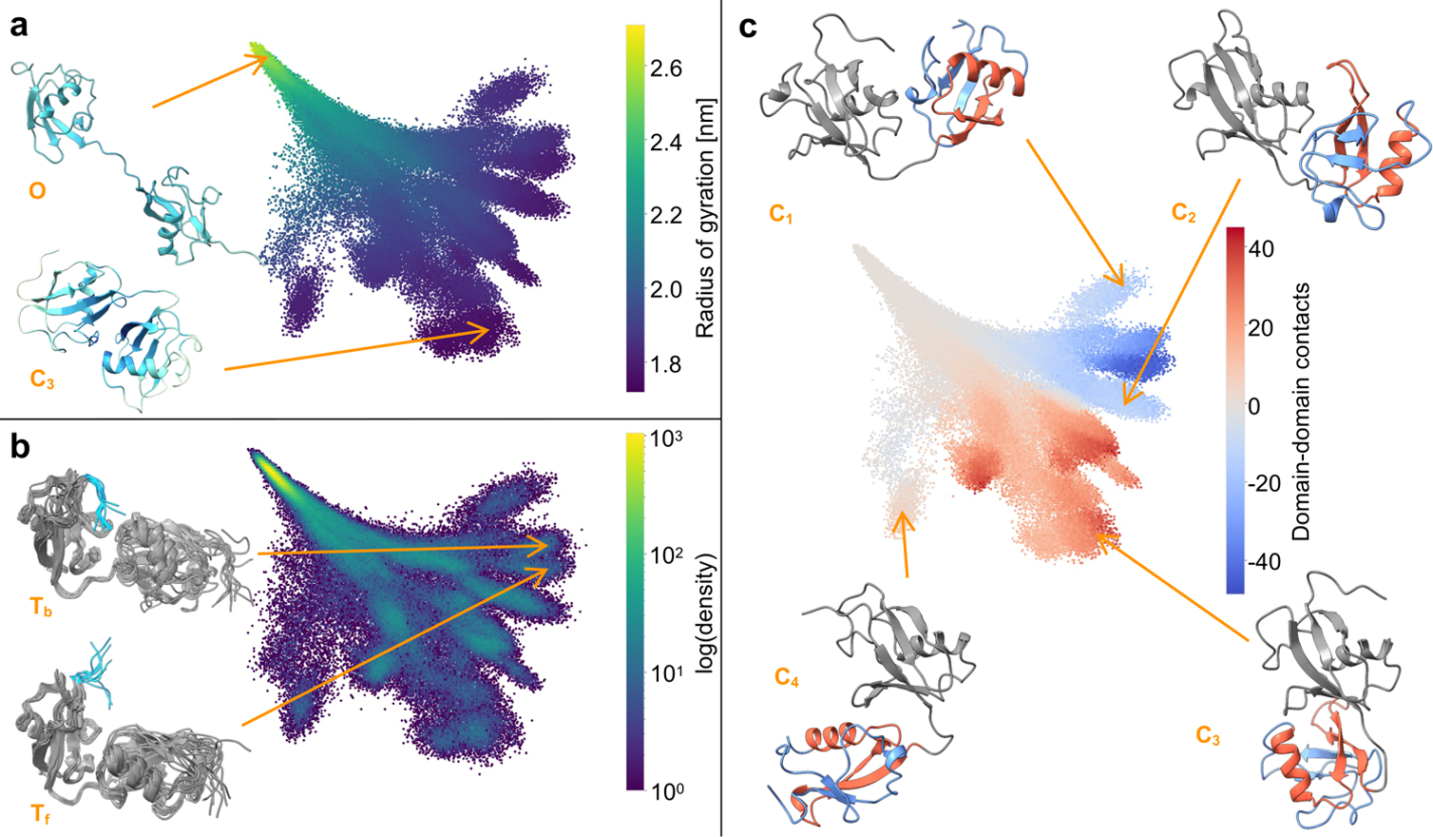
Map of residue interaction landscape of FAT10: (a) Colored by radius of gyration, representative open (O) and closed (C_3_) conformations are shown, colored by closeness centrality, (b) colored by density with representative bundles of conformations with the C-terminal tail bound T_b_ and free T_f_, and (c) colored by domain–domain contacts and shown with representative conformations. Red means many contacts of the gray C-terminal domain with the red section of the N-terminal domain, blue means many contacts with the blue section, and gray means low contact count overall or equal contacts with red and blue section).

To visualize the conformational dynamics of FAT10 over all 50 simulation trajectories, the 165-dimensional closeness vector of all simulation frames was projected into a two-dimensional map of the residue interaction landscape, colored by different structural properties (see Figure 5). Coloring by the *R*_*g*_, Figure 5a shows that there is a clear separation along the map’s first diagonal axis, between low-contact, open states at the top left of the map and high-contact, closed states at the bottom right. There is a substantially higher number of distinct high-contact states than for Trp-Cage. They differ in the interfaces between the two domains of FAT10. In the map, they are finely separated along the second diagonal from the bottom left to the top right, based on the noncovalent contacts at the domain interface. This is shown in Figure 5c. Here, the map is colored by the contacts of the C-terminal domain with different sections of the N-terminal domain: red means many contacts of the C-terminal domain (colored in gray in the representative structures in Figure 5c C_1_–C_4_) with the red section of the N-terminal domain, blue means many contacts with the blue section, gray means low contact count overall or equal contacts with red and blue section. At an even finer level, the map resolves the dynamic behavior of the flexible regions of FAT10. As an example of this, two closed states of FAT10 are shown in Figure 5b. The states share a similar relative orientation of the domains. However, in one state T_f_, the C-terminal tail moves freely, exposed to the solvent, while in the other state (T_b_), the C-terminal tail is in close contact with the rest of FAT10, shielded from solvent exposure and forming a part of the domain interface. Whether FAT10 is open or closed, how its domains interact and how its flexible regions behave are crucial determinants of the function of FAT10. FAT10 has a plethora of protein–protein interactions^24,49^ and different closed conformations offer different interaction sites for binding proteins. The N-terminal and C-terminal tails are essential for binding to the proteasome and the substrate, respectively. Deletion mutants for the flexible linker display complete loss of activity.^46^ A map that resolves these conformational dynamics of this protein while retaining an interpretable global structure is a useful tool for understanding the high-dimensional MD data on this complex protein system.

## 3 Discussion and Conclusions

Describing proteins as graphs is a natural and powerful way to capture their three-dimensional structure. By projecting the graphs from a protein MD trajectory into a low-dimensional embedding, we obtain a map of the protein’s residue interaction landscape, providing a mesoscopic view of its conformational ensemble. Here, we have shown that the closeness fingerprint offers a compact and expressive feature set to perform the graph embedding with the dimensionality reduction algorithm EncoderMap. The resulting workflow for a feature-based, temporal graph embedding for RINs can be generalized to different protein systems without system-specific prior input. We have shown this for two different protein systems with different sizes and different conformational behaviors: the 20-residue fast-folding protein Trp-Cage and the 165-residue multidomain protein FAT10.

In principle, any protein can be translated into a graph, making the presented workflow easily generalizable to different proteins. Due to its modularity, it can incorporate any type of protein graph that allows for the calculation of node centralities. There are many different tools and formalisms to translate proteins into graphs,^13,14,16^ making it possible to adjust the information content of the description, for example, by including details on the interaction types^50^ or energies^17^ or by describing the protein at higher (atomistic)^51^ or lower (coarse-grained)^16,20^ resolution. It is important that the formalism for the protein-graph translation is chosen appropriately for the system under study and the underlying scientific question.^52^ Transforming the Cartesian coordinates of the protein into a relational data set of its interactions is bound to reduce the information content in the resulting description. For example, local details in the dynamics of otherwise rigid, globular proteins may get lost in the transformation. This means that the presented workflow is most suited for situations where protein systems undergo strong changes in their residue interactions.

Due to the noneuclidean nature of graph data structures, applying machine learning algorithms on graphs is challenging. Recently, there has been a surge of various deep learning approaches, in particular graph neural networks, being investigated to learn suitable representations of graph structured data.^53,54^ In many settings, these approaches have to accommodate graphs of different sizes and unknown node identities, for example, in representation learning for small molecules.^55−57^ In contrast, our specific task of embedding temporal graphs from protein MD trajectories is more focused, making it possible to rely on a deterministic graph representation and forgoing a training or message passing process. The graphs from the RINs have a fixed size, a known node identity and a known node ordering naturally defined by the protein sequence. This means we can take advantage of using node-specific features as a graph representation for further downstream processing.

The closeness fingerprint used here as the node feature set has several properties that make it suitable for the use as an RIN graph representation and in our view also as a general feature set for proteins: Like other featurizations of protein conformations, the closeness fingerprint is a set of internal features, invariant to rotation and translation. It is a simple metric that is implemented in various network analysis tools.^35,58^ Its clear mathematical definition (requiring no training) and close correspondence to distance-based featurizations in Cartesian space (“how close is the residue to all other residues?”) make it a robust protein description with a high *ante-hoc* interpretability.^59^ This robustness and interpretability set the closeness fingerprint apart from other RIN representations using graph neural networks or other centrality metrics.^18,60^ Falling between local feature sets like backbone dihedral angles and global metrics like the fraction of native contacts, the closeness fingerprint from protein RINs offers a mesoscopic perspective on protein conformation. For illustration, embeddings for FAT10 using a number of local feature sets are included in the Supporting Information (Figure S2). Because the closeness centrality for each residue takes into account the paths from that residue to all other residues, it connects local information on the neighborhood of the individual residues with global information on the contact topology of the entire protein. With this, the closeness fingerprint extracts a compact, *N*-dimensional feature set from the full (*N* × *N*-dimensional) residue–residue contact map.^15,32^

We retain the mesoscopic character of the closeness fingerprint by embedding it with EncoderMap. Combining a neural network autoencoder with a tunable Euclidean distance cost function, it offers a nonlinear, MDS-like behavior with the ability to handle large amounts of data. This allows us to set a focus on intermediate distances and to use the closeness fingerprint for a mesoscopic graph distance metric as proposed by Donnat and Holmes.^32^ This would be different using a global embedding like principal component analysis or more local algorithms like UMAP^61^ (Figures S3 and S4). The fact that this mesoscopic landscape embedding retains a global structure and is constructed with a focus on residue interactions means that we can qualitatively interpret the dimensions of the map and extract information about the relationships between structures from different map regions. This can come at the cost of losing fine structural resolution and discrete, local clustering. For example, different low-contact (e.g., unfolded/open) structures that may be separated, e.g., in dihedral space, but do not differ with respect to their residue interactions, will not be separated in the map of the residue interaction landscape. They will be tightly grouped in a region that can be recognized as a low-contact region. This means that one needs to be cautious when applying clustering algorithms directly to the low-dimensional embedding.

Rather than resolving the fine, local details in protein structures, the strength of the presented approach is to provide a coherent, interpretable visualization of a protein’s residue interaction landscape. By combining it with an algorithm dedicated to finely resolved structure-based clustering,^62^ one could build a tool to separate, aggregate, and select protein conformations from a heterogeneous ensemble for further, more detailed downstream analyses based in graph theory. This could include investigations of allostery,^22,63,64^ community detection algorithms^65^ or graph spectral methods.^66^ It could also augment kinetic models, such as finely resolved Markov state models,^67^ with a visualization method. Because the graph formalism is such a flexible protein representation, the could also be used in scale-bridging applications to improve sampling based on the low-dimensional embedding.^68^ Since the presented method is purely based on graph topology, it could also be suited to visualize the interaction dynamics of other complex dynamic systems that can be described by networks.^69,70^

## 4 Methods

### 4.1 Molecular Dynamics Simulations

#### 4.1.1 Trp-Cage

The 208 μs long folding simulation of Trp-Cage (K8A mutant of the thermostable Trp-Cage TC10b) as reported in ref (23) has been kindly provided by D. E. Shaw research. The trajectory had been obtained on the Anton supercomputer using the CHARMM22 force field and a modified TIP3P water model. We used the full simulation trajectory, sampled every 200 ps with over 1,000,000 frames for our analysis.

#### 4.1.2 FAT10

Human leukocyte antigen (HLA)-F adjacent transcript 10 (FAT10) was simulated atomistically in full length and with a procedure similar to that in Aichem et al.^46^ 50 simulations were performed for 50 ns each, with 25 different starting conformations of FAT10 and 2 different ion concentrations (no additional ions and 150 mmol NaCl, physiological NaCl concentration). The starting structures of FAT10 were constructed from the two separate, experimentally solved protein data bank (PDB) structures of the two domains.^46^ Chain B from the crystal structure 6GF1 was used for the N-terminal domain (ND), and chain 1 from the NMR (nuclear magnetic resonance) structure bundle 6GF2 was used for the C-terminal domain (CD). Both were mutated to restore their wild-type sequence. The two domains were subsequently joined in a maximally extended conformation. In the resulting fully extended molecule, the ψ angle in residue D85 was rotated 360° in 25 steps to produce the 25 starting structures. All MD simulations for FAT10 were carried out using the GROMACS simulation package (version 2018.5)^71^ with the GROMOS96 54/A7 force field^72^ and the SPC/E water model.^73^ The temperature was kept at 300 K with a velocity rescaling thermostat^74^ and the pressure at 1 bar with the Parinello-Rahman barostat.^75^ The default md leap-frog integrator was used with a time step of 2 fs and a cutoff for van der Waals interactions of 1.4 nm. Bonds were constrained using the LINCS algorithm^76^ and electrostatic interactions were treated with the particle mesh Ewald scheme^77^ and a cutoff of 1.4 nm. Protonation states according to a pH of 7 were used. The starting structures were placed into a dodecahedral simulation box (box vector: 12.5, 12.5, 10.5 nm) with periodic boundary conditions. An energy minimization was performed in vacuum for 100 ps before solvation and the addition of ions. Six Cl^–^ ions were added for all simulations for neutralization. An additional 119 Na^+^ and Cl^–^ ions were added for 25 of the 50 simulations to achieve an approximately physiological NaCl concentration of 150 mmol. After this, another energy minimization was done with position restraints for 100 ps. Here, for the simulations with additional NaCl, a time step of 1 fs was chosen for the energy minimization steps. Equilibration was done in three simulation runs of 100 ps, first under constant temperature (NVT) with position restraints, then under constant pressure (NpT) with position restraints and again under constant pressure (NpT) without position restraints. The potential energy and temperature during the final equilibration runs were checked to ensure stability. Production simulation runs were performed for 50 ns each, totaling 2.5 μs of simulation time. After reducing the number of frames of each resulting trajectory to 5001 (resulting in a temporal resolution of 10 ps/frame), the protein was centered, made whole and checked for secondary structure instabilities using functions of the GROMACS suite. The resulting simulation data set consisted of 50 trajectories with a total of 250,050 simulation frames. It should be noted that the presented data does not (yet) represent a fully sampled, atomistic phase space of FAT10. It does offer a wide range of interactions between two loosely connected folded protein domains and additional intrinsically disordered protein regions, which is why we chose the system and this data set as an example.

### 4.2 Calculation of Closeness Centralities

Let *G*(*V*, *E*) be a graph of the protein residue interaction network. The graph consists of a set of nodes (vertices) *V* with size *N* = |*V*|, the number of residues in the protein, and a set of edges (interactions) *E* of size *M* = |*E*|. Each node *v*_*i*_ in *V* represents one amino acid in the protein sequence, and each edge *e*_*i*,*j*_ in *E* represents an interaction between two amino acids. The closeness centrality *c*_*i*_ of each node *v*_*i*_ is then given by the reciprocal of the mean shortest path length *d*(*v*_*i*_, *v*_*j*_) from that node to all other nodes *v*_*j*≠*i*_, normalized by the number of nodes *N*:
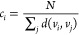
1Since the edges of the graph are unweighted, i.e., they have weight 1, the shortest (geodesic) path length *d*(*v*_*i*_, *v*_*j*_) is an integer count of the lowest number of edges separating *v*_*i*_ and *v*_*j*_. Here, a simple geometric criterion is used to determine the residue interaction: Two nodes are connected by an edge, if the smallest distance between any atoms of the two corresponding amino acids (as calculated by the function compute-contacts with them minimum distance criterion in the python package mdtraj)^78^ is below a cutoff of 6.0 Å, with the exception of interactions between directly neighboring residues in the protein sequence. We have found the approach to be fairly robust toward the choice of the cutoff, when the graphs stay connected. Nonetheless, the method of RIN construction is a free parameter in the presented approach and should be chosen with care.^52^ The graphs were constructed using the python package NetworkX.^35^ The closeness centrality was calculated using the python package NetworKit.^58^ The *N*-dimensional vector of the *N* closeness centralities *c*_*i*_, ordered by the amino acid sequence of the protein, then forms the closeness fingerprint describing each protein conformation from the simulation trajectory. It was transformed to be in a range of 0–1 by dividing by its maximum over all simulation frames. The fact that the RINs of proteins studied here have a relatively low number of nodes and the high-performance implementation of the closeness centrality algorithm in NetworKit means that this fairly demanding calculation only takes on the order of a few minutes on a desktop computer with an Intel Core i7-8700K 12 core 3.70 GHz CPU for the large data sets investigated here. For very large proteins, where scaling might become an issue, NetworKit also offers an approximate closeness centrality algorithm with better scaling behavior. We want to note that other path-based centrality metrics such as betweenness centrality or eigenvector centrality^18,60^ could provide a suitable picture of protein behavior, when projected into a low-dimensional embedding, likely highlighting other aspects of a protein’s dynamics.

### 4.3 Embedding with EncoderMap

The *N*-dimensional closeness fingerprint was used as input for the nonlinear dimensionality reduction algorithm EncoderMap.^79,80^ EncoderMap combines a neural network autoencoder with a sigmoid cost function to produce a meaningful low-dimensional representation of a high-dimensional feature space. It consists of an encoder part with an input layer and several hidden layers, a bottleneck layer with few neurons, and a decoder part with several more hidden layers and an output layer. It is trained to accurately reproduce the information from the input layer in the output layer. Because it has to pass the information from the input through the bottleneck layer before decoding it into the output, it learns to encode it into a low-dimensional representation of the inputs. This learned mapping or code can then be used to project the points from high-dimensional space into the low-dimensional map. In addition to the cost function of the autoencoder *C*_*auto*_, which reflects the difference between the input and the output, there is an additional pairwise distance cost function *C*_*sketch*_, which forces the autoencoder to arrange the points in the low-dimensional map so that the arrangement of points in low-dimensional space reflects the distances between the points in high-dimensional space:
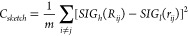
2where *m* is the number of projected frames and *R*_*ij*_ and *r*_*ij*_ are the pair distances between the frames in the high- and low-dimensional space, respectively. Using the sigmoid function:
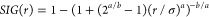
3enables one to suppress the impact of small distances between very similar points and of very large distances that would be hard to reproduce accurately in low-d space. It is adjusted with the parameters σ, *a* and *b*. It was found that the qualitative results of several EncoderMap runs with different parameters were fairly robust toward parameter selection. The parameters (shown in Table 1) were thus chosen by visual inspection to produce residue interaction landscapes with a high resolution of structural detail. All other parameters of EncoderMap were left at the default settings.^79^ The sigmoidal transformation to pairwise distances was first applied in the multidimensional- scaling-like algorithm Sketch-map^33^ and adapted for EncoderMap. In EncoderMap, the distance cost between the vectors in the high-dimensional space is calculated as the Euclidean distance. This means that the distance metric used to arrange the graphs in low-d embedding space is the Euclidean distance between their node centralities. There are many other ways to calculate distances between graphs, such as the graph edit distance,^81^ the Hamming distance,^82^ the Jaccard distance^83^ or spectral distances,^84^ and they differ in the way in which they capture local and global changes in graph topology. It has been shown that Euclidean distances between centralities of graphs are suitable distance metrics to capture local and global (“gLocal”) changes in a graph topology,^32^ which is why they are applied here. Should a different focus be desired, the closeness fingerprint can in principle serve as input for other embedding algorithms. We have included embeddings for Trp-Cage and FAT10 obtained by the more global principal component analysis and the more local UMAP algorithm in the Supporting Information (Figures S3 and S4).

**Table 1:** Parameters for EncoderMap

Protein system	Trp-Cage	FAT10
Learning rate	0.00001	0.00001
Regularization const.	0.00001	0.00001
Periodicity	*∞*	*∞*
*N*_frames_	1044000	250500
*N*_steps_	20000	50000
Sigmoid parameters: σ_*h*_, *a*_*h*_, *b*_*h*_, σ_*l*_, *a*_*l*_, *b*_*l*_	0.5, 6, 6, 1, 2, 6	1.0, 6, 6, 1, 2, 6

### 4.4 Data Analysis and Visualization

All data analysis tasks were performed using Python 3. General computations were performed using NumPy^85^ and pandas.^86^ Structural analyses, such calculations of RMSD, *R*_*g*_ and distances were performed using MDTraj.^78^ The contacts between FAT10s domains were calculated by separating the CD into two sections (red and blue) at residue 124 and summing up the number of contacts, determined by the above distance criterion, of the ND with the respective sections of the CD. The two contact counts were combined by counting the contacts with the blue section negatively. Data visualization was performed using Matplotlib,^87^ plotly and plotly dash. Visualization of molecular structures was performed using ChimeraX.^88,89^

## Supporting Information Available

Comparison of individual closeness fingerprints of FAT10, Encodermap embeddings of other feature sets for FAT10, alternative embeddings of the closeness fingerprint for Trp-Cage and FAT10 (PDF)
