# Supplementary material for: Visualizing the Residue Interaction Landscape of Proteins by Temporal Network Embedding
Source: J Chem Theory Comput. 2023 Apr 26;19(10):2985–95. doi: 10.1021/acs.jctc.2c01228 (PMC10210247; doi:10.1021/acs.jctc.2c01228)
Supplement: Supplementary file 1 — ct2c01228_si_001.pdf [file ct2c01228_si_001.pdf]

SUPPORTING INFORMATION: Visualizing the residue interaction landscape of proteins by temporal network embedding

Leon Franke[*,†,‡] and Christine Peter[*,†]

†*Department of Chemistry, University of Konstanz, Universitätsstraße 10, Konstanz 78457, Germany*

‡*Konstanz Research School Chemical Biology*

E-mail: Leon.Franke@uni-konstanz.de; Christine.Peter@uni-konstanz.de

S-I Comparing closeness fingerprints

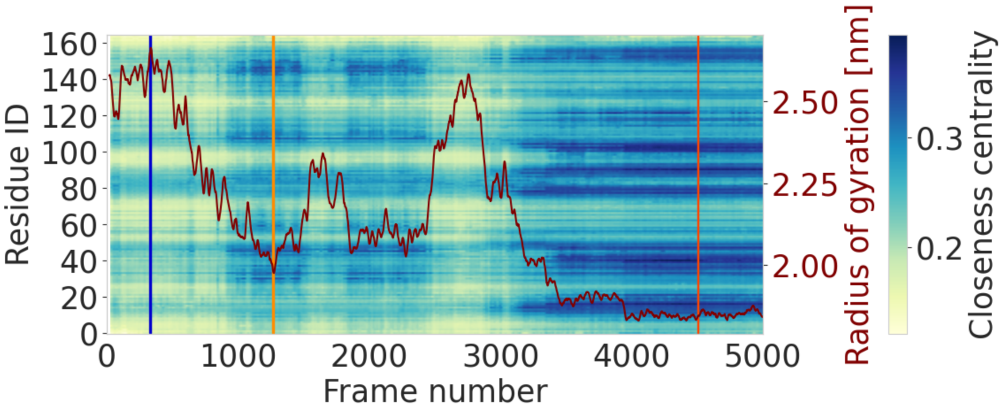

(a) Closeness fingerprint and radius of gyration (R_g, red) for FAT10 over one simulation trajectory, selected frames are marked by colored bars.

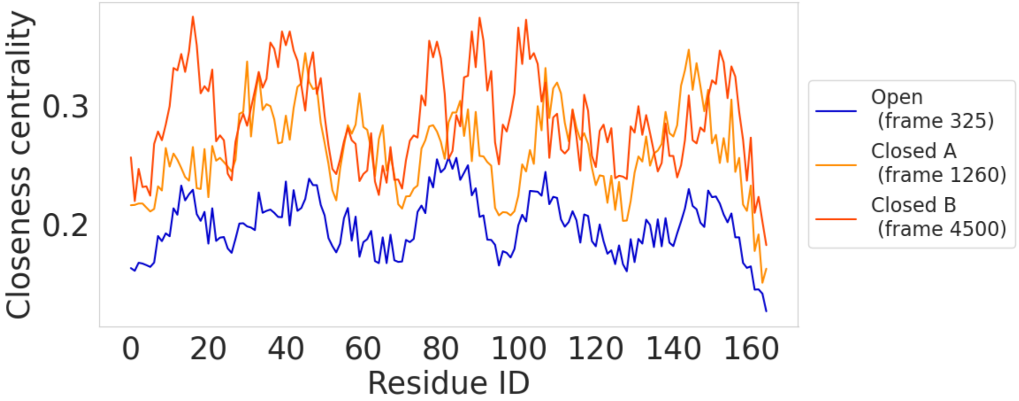

(b) Closeness fingerprints of selected frames from one simulation of FAT10 shown in panel a.

Figure S1: To show how the closeness fingerprint integrates information on the global protein conformation and the local residue environment, we show the closeness fingerprint of three individual conformations from one simulation of FAT10. In an open, low-contact, (high R_g) conformation (blue), in which the two domains have no non-covalent interactions, the paths across the residue interaction network (RIN) are long. This leads to an overall lower closeness centrality for all residues. In the closed (low R_g) conformations (orange and red) the bridging non-covalent domain-domain contacts between the two domains reduce the path lengths through the entire RIN, increasing the closeness centrality globally for all residues. Each residue "notices" that the protein has closed, even if it is not directly at the domain interface. Yet, each residue still carries fine-grained information about its local neighborhood (influenced e.g. by secondary structures, a salt bridge, or a domain-domain contact) encoded in its closeness centrality. The resulting characteristic closeness fingerprint for each RIN makes it possible to distinguish e.g. the two closed conformations of FAT10 with different domain-domain interactions (orange and red).

S-II Alternative featurizations for FAT10

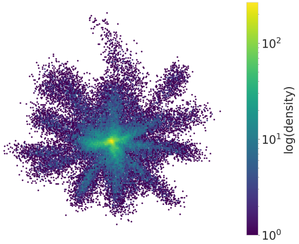

(a) Encodermap of FAT10 based on the contact map (adjacency matrix), colored by density (reduced data due to memory constraints).

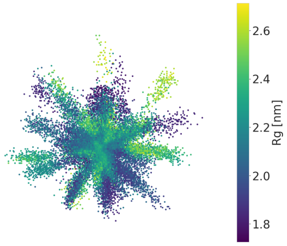

(b) Encodermap of FAT10 based on the contact map (adjacency matrix), colored by R_g (reduced data due to memory constraints).

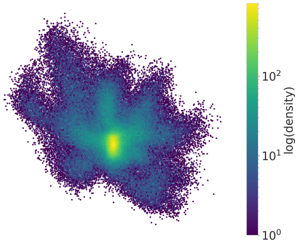

(c) Encodermap of FAT10 based on residue-wise contact counts (degree centrality), colored by density.

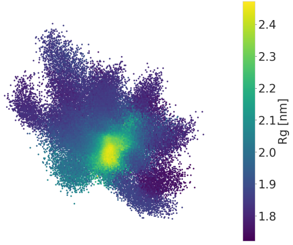

(d) Encodermap of FAT10 based on residue-wise contact counts (degree centrality), colored by R_g.

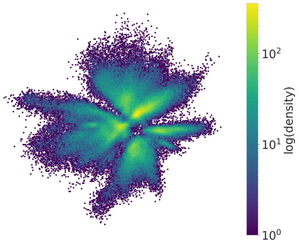

(e) Encodermap of FAT10 based on backbone dihedral angles, colored by density.

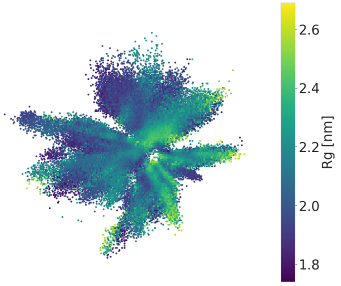

(f) Encodermap of FAT10 based on backbone dihedral angles, colored by R_g.

Figure S2: To demonstrate the mesoscopic nature of the closeness fingerprint, we show Encodermap embeddings of other commonly used feature sets for FAT10. The discretized and flattened contact map or adjacency matrix, the contact count or degree centrality for each residue and the dihedral angles of the protein backbone are local features that do not incorporate global information on the full protein conformation. That is, a local residue feature only changes if that specific residue is directly involved in a conformational change. We observe that the resulting embeddings have a mostly circular shape, lacking a globally interpretable structure. Separating low-contact (high R_g) from high-contact (low R_g) structures and distinguishing different high-contact conformations is difficult based on the local maps and fairly straightforward with a mesoscopic map (Figure 5 in the manuscript).

S-III Alternative embeddings

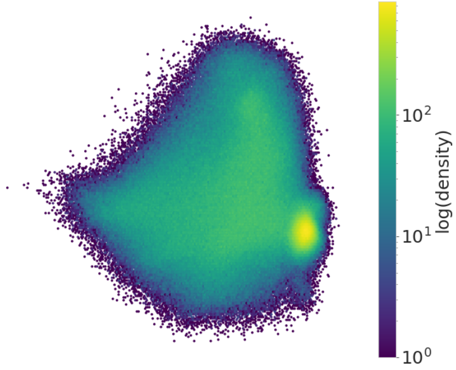

(a) Principal component analysis (PCA) for closeness fingerprint of Trp-Cage, first two PCs, colored by density.

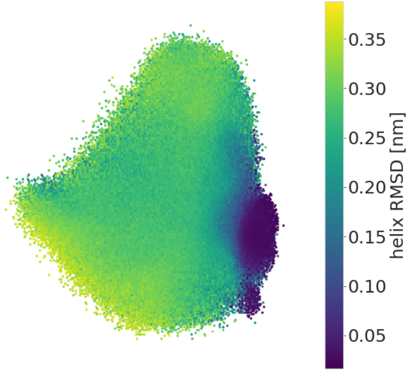

(b) PCA for closeness fingerprint of Trp-Cage, first two PCs, colored by RMSD from folded helix (residues 2-8).

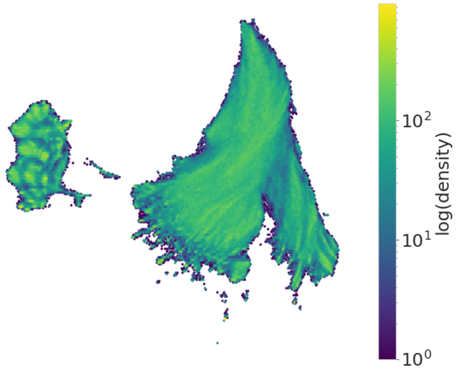

(c) UMAP embedding for closeness fingerprint of Trp-Cage, colored by density.

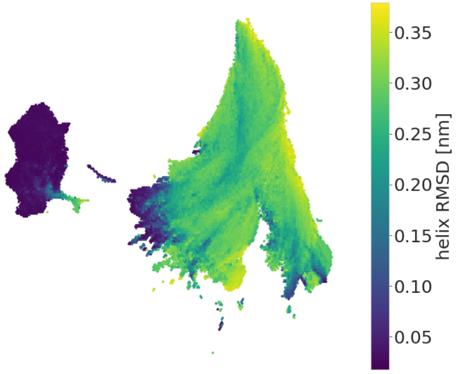

(d) UMAP embedding for closeness fingerprint of Trp-Cage, colored by RMSD from folded helix (residues 2-8).

Figure S3: Embedding the closeness fingerprint of Trp-Cage with different algorithms allows us to assess the impact of embedding algorithms with a more global (PCA) or more local (UMAP) tendency. The PCA gives an interpretable, coherent picture of the dynamics of Trp-Cage, very similar to Encodermap, albeit making the diffusion-collision folding pathway (characterized by a low helix RMSD) somewhat less discernible. The UMAP embedding appears less coherent and less interpretable with respect to the helix RMSD and tears apart a folded region from the rest of the map.

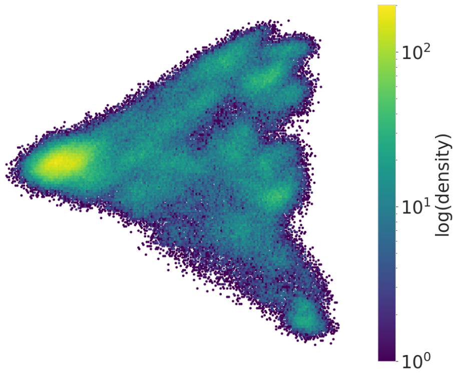

(a) PCA for closeness fingerprint of FAT10, first two PCs, colored by density.

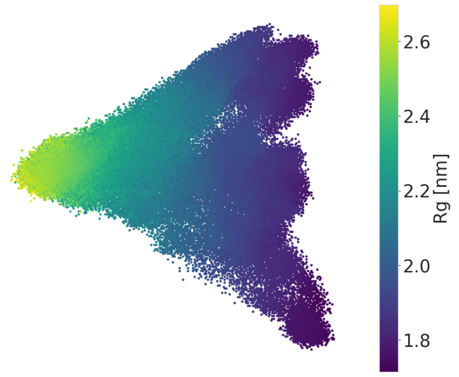

(b) PCA for closeness fingerprint of FAT10, first two PCs, colored by R_g.

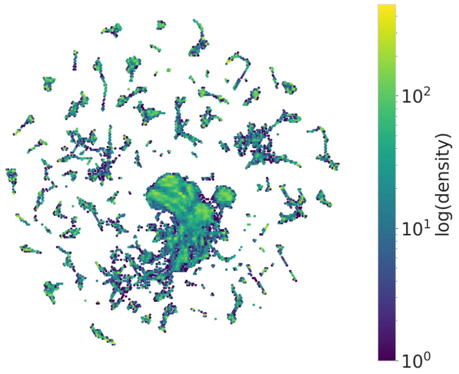

(c) UMAP for closeness fingerprint of FAT10, first two PCs, colored by density.

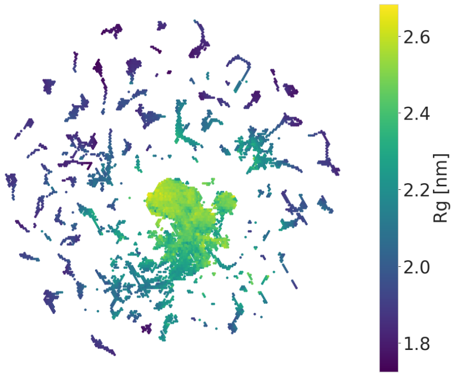

(d) UMAP for closeness fingerprint of FAT10, first two PCs, colored by R_g.

Figure S4: Embedding the closeness fingerprint of FAT10 with different algorithms with a more global (PCA) or more local (UMAP) tendency. The PCA-embedding for the closeness fingerprint of FAT10 shows a the global nature of the linear embedding provided by PCA: While a coherent global structure is retained, the resolution of the map is reduced in comparison to the Encodermap embedding. This makes it difficult to distinguish different closed conformations of FAT10 and limits further down-stream processing. The UMAP embedding shows UMAP's focus on reproducing local structure, which can tear clusters apart and can lead to a somewhat circular and less coherent map that makes it harder to make out the relationships between embedded frames.
